# An ATF3-CreERT2 Knock-In Mouse for Axotomy-Induced Genetic Editing: Proof of Principle

**DOI:** 10.1523/ENEURO.0025-19.2019

**Published:** 2019-04-09

**Authors:** Seth D. Holland, Leanne M. Ramer, Stephen B. McMahon, Franziska Denk, Matt S. Ramer

**Affiliations:** 1 International Collaboration on Repair Discoveries, the University of British Columbia, Vancouver, British Columbia, Canada V5Z 1M9; 2 Biomedical Physiology and Kinesiology, Simon Fraser University, Burnaby, British Columbia, Canada V5A 1S6; 3 Wolfson Centre for Age-Related Diseases, King’s College London, London, United Kingdom SE1 1UL

**Keywords:** Activating Transcription Factor 3, gene editing, peripheral nerve, Schwann cells, sensory neurons, spinal cord injury

## Abstract

Genome editing techniques have facilitated significant advances in our understanding of fundamental biological processes, and the Cre-Lox system has been instrumental in these achievements. Driving Cre expression specifically in injured neurons has not been previously possible: we sought to address this limitation in mice using a Cre-ERT2 construct driven by a reliable indicator of axotomy, activating transcription factor 3 (ATF3). When crossed with reporter mice, a significant amount of recombination was achieved (without tamoxifen treatment) in peripherally-projecting sensory, sympathetic, and motoneurons after peripheral nerve crush in hemizygotes (65–80% by 16 d) and was absent in uninjured neurons. Importantly, injury-induced recombination did not occur in Schwann cells distal to the injury, and with a knock-out-validated antibody we verified an absence of ATF3 expression. Functional recovery following sciatic nerve crush in ATF3-deficient mice (both hemizygotes and homozygotes) was delayed, indicating previously unreported haploinsufficiency. In a proof-of-principle experiment, we crossed the ATF3-CreERT2 line with a floxed phosphatase and tensin homolog (PTEN) line and show significantly improved axonal regeneration, as well as more complete recovery of neuromuscular function. We also demonstrate the utility of the ATF3-CreERT2 hemizygous line by characterizing recombination after lateral spinal hemisection (C8/T1), which identified specific populations of ascending spinal cord neurons (including putative spinothalamic and spinocerebellar) and descending supraspinal neurons (rubrospinal, vestibulospinal, reticulospinal and hypothalamic). We anticipate these mice will be valuable in distinguishing axotomized from uninjured neurons of several different classes (e.g., via reporter expression), and in probing the function of any number of genes as they relate to neuronal injury and regeneration.

## Significance Statement

Understanding reactions to neurotrauma and overcoming obstacles to neural regeneration should benefit from the ability to genetically label or otherwise edit the genome of injured neurons. We sought to achieve this in mice by driving Cre recombinase expression under the control of activating transcription factor 3 (ATF3), which is robustly induced by axotomy in several populations of peripheral and central neurons. When crossed with reporter mice, recombination occurred only in injured neurons following sciatic nerve injury or spinal hemisection. Peripheral nerve injury (PNI)-induced neuronal phosphatase and tensin homolog (PTEN) excision also resulted in improved regeneration and more complete functional recovery. These results demonstrate the feasibility and utility of axotomy-induced recombination and represent a new tool for investigating genetic control of injury responses and regeneration.

## Introduction

Advances in genome editing techniques have created opportunities to dissect out the function of specific genes and their contributions to health and disease. Among the most widely used of these tools is the Cre-Lox system, in which Cre expression can be restricted spatially, to a single tissue or population of cells through insertion under a specific promoter (Wagner et al., 1997; Clausen et al., 1999; Agarwal et al., 2004), or temporally, by fusing the protein to a mutated estrogen receptor selective for tamoxifen (Indra et al., 1999; Hayashi and McMahon 2002; Leone et al., 2003). In neurotrauma, the combination of spatial and temporal control of gene expression has facilitated the identification of the function of individual neural cells (Sainsbury et al., 2002; Q. Liu et al., 2010), and manipulated their phenotype to promote repair of the damaged nervous system (Park et al., 2008; Sun et al., 2011). A limitation in applying Cre-Lox to investigations of neurotrauma is the inability to selectively express Cre in populations of neurons affected by unique pathologies, such as tau protein-associated degeneration, immune-mediated degradation, or simple axotomy. This could notionally be achieved by taking advantage of a gene that is highly expressed only when the neuron is degenerating or axotomized. Pathology-induced recombination would be of use to both identify the affected cell populations and to edit those populations’ genetic makeup. Here, we present a novel transgenic approach to effecting genome editing on axotomy via an injury-specific gene.

Activating transcription factor 3 (ATF3) is a transcription factor that belongs to the basic leucine zipper family (Liang et al., 1996). ATF3 is an immediate early gene; its transcription is initiated extremely rapidly following the appropriate stimulus. ATF3 is considered a reliable marker of neuronal somata with injured peripheral axons (Tsujino et al., 2000; Mason et al., 2003); ATF3 mRNA can be detected after peripheral axotomy as early as 6 h after injury (Tsujino et al., 2000) and can achieve a 130-fold increase 72 h after axotomy (Seijffers et al., 2007). Other genes upregulated following injury include c-Jun and GAP-43 (Tetzlaff et al., 1991; Broude et al., 1997), but none are upregulated as fast and prominently as ATF3, making it the most reliable marker of peripheral nervous system (PNS) injury. These changes in gene expression constitute part of the pro-regenerative “cell body response” to injury. A loss of ATF3 function has been shown to reduce the regeneration observed after a peripheral nerve injury (PNI; Gey et al., 2016).

Damage to the PNS is a common outcome of motor vehicle accidents, penetrating trauma, and falls (Kouyoumdjian, 2006). The PNS has the ability to regenerate injured axons that result in full functional recovery: however, despite the theoretical regenerative capacity of the PNS, clinical PNI often results in permanent disability, since regeneration is often incomplete when the distance from the axotomy to the soma is far, the gap between distal and proximal stumps is large, or the time until surgical intervention is long. Reports suggest that ∼5% of all patients admitted to a level 1 trauma center sustained a PNI (Noble et al., 1998), highlighting the need for strategies that augment this inadequate regenerative response.

Phosphatase and tensin homolog (PTEN) is an inhibitor of the PI3K/AKT/mTOR cell growth and proliferation pathway (Stambolic et al., 1998). The loss of PTEN function *via* viral Cre transfection induces a regenerative response in corticospinal neurons after a spinal cord injury (K. Liu et al., 2010), a finding that has been reported in other CNS axonal injury models (Park et al., 2008). In the PNS, in which regeneration is more robust but still suboptimal, PTEN deletion has been shown to have modest augmentative effects on axonal regeneration (Gallaher and Steward, 2018). This allows for the unique opportunity to compare a pathology-dependent Cre upregulation strategy with a more traditional viral Cre transfection model.

Here, we present a novel transgenic mouse model that allows for the selective genetic editing of injured neurons via the insertion of a Cre-ERT2 construct under the native ATF3 promoter. We confirm expression of the Cre-ERT2 construct in injured neurons after PNI using a fluorescent reporter line and we demonstrate that this model may also be used to effect recombination in select populations of axotomized CNS neurons. Finally, we show that neuronal injury-specific Cre-ERT2 expression can be used to functionally alter the regenerative capacity of neurons though excision of crucial PTEN exons, a proof-of-principle experiment that illustrates the utility and potential of this model in neurotrauma.

## Materials and Methods

### Animals

All animal procedures were performed in accordance with the University of British Columbia and King’s College London animal care committees’ regulations. All mice were between two and four months of age and equally distributed between sexes.

The ATF3-CreERT2 (ATF3^cre^) strain used was generated and described by Denk et al. (2015). Briefly, the Cre-ERT2 construct was inserted directly after the ATG start codon of the second ATF3 exon followed by a 3’ untranslated region and a polyadenylation tagging sequence. For some experiments, this line was subsequently crossed to a floxed stop tdTomato Ai14 reporter line (RRID:IMSR_JAX:007908; Madisen et al., 2010). The ATF3^cre^ line was also crossed with conditional PTEN deletion line (PTEN^fl/fl^) with LoxP sites flanking exon 5 of the PTEN gene (RRID:IMSR_JAX:006440; Lesche et al., 2002; in some cases also crossed with the Ai14 line to determine recombination efficiency). Mice were maintained on a mixed C57BL/6J x 129SvEv background. ATF3-CreERT2 mice are available by request from the laboratories of Franziska Denk (King’s College London) and Matt Ramer (the University of British Columbia).

### Surgical procedures

For the sciatic nerve crushes the animals were administered buprenorphine (0.02 mg/kg; Temgesic) and ketoprofen (5 mg/kg; Anafen) subcutaneously for prophylactic analgesia. Once anaesthetized with isoflurane (5% induction, 2–3% maintenance; Fresenius Kabi Canada Ltd.), the sciatic nerve was exposed by blunt dissection and crushed for 15 s (thrice, 5 s each) with fine #5 forceps at the sciatic notch. For the brachial nerve crush the same anesthetic and analgesic protocols were used. The distal brachial plexus was exposed in the upper forelimb, the median radial, and ulnar nerves were crushed with fine forceps. Tamoxifen (Sigma) was dissolved in wheat germ oil (Denk et al., 2015) and injected at a concentration of 75 mg/kg at the time of injury. Pure anti-estrogen ICI 182780 (Tocris) was delivered by gavage the day before, the day of, and the day after injury (20 μg dissolved in sunflower oil). For the 2-d regeneration assays, the injury site was marked with forceps dipped in graphite (ThermoFisher). For retrograde tracing, 1 μl of 5% Fluorogold (Santa Cruz Biotechnologies) dissolved in 50:50 DMSO:PBS (Sigma-Aldrich) was injected intraneurally immediately before injury with a 10-μl Hamilton syringe (Sigma-Aldrich).

Spinal hemisection was also conducted with the same anesthetic and analgesic protocols outlined above. A midline incision was made over the lower cervical/upper thoracic spinal cord, and the C8 and T1 laminae were removed. A 25-gauge needle was inserted dorso-ventrally at the midline between the C8 and T1 spinal segments to allow relatively atraumatic entry of one blade of a pair of microscissors. The cord was laterally transected with microscissors; after hemostasis was established, the muscle and skin were closed in layers with sutures. Mice were killed 7 d later.

### Functional outcomes

To investigate the following anatomic and functional recovery outcomes we employed a transgenic line with only the *Atf3* and *Pten* alleles altered (i.e., without reporter to avoid potential confounds associated with high tdtomato expression). Specifically, in the first set of experiments we compared ATF3^+/+^, ATF3^+/cre^, and ATF3^cre/cre^ mice to determine similarities to previous knock-out models (Gey et al., 2016). In the second, set we compared ATF3^+/cre^:PTEN^+/+^ mice with ATF3^+/cre^:PTEN^fl/fl^ to compare axotomy-induced PTEN deletion with virally-mediated PTEN deletion (Gallaher and Steward, 2018).

### Behavioral testing

Reflex withdrawal or crossed extension (i.e., a nocifensive response) on strong toe pinch was assessed in mice lightly anesthetized with 2% isoflurane. On loss of righting reflexes, each animal was removed from the induction chamber and placed prone on a table. To confirm light anesthesia, the base of a contralateral toe was pinched with curved serrated forceps. If there was no initial response, the other toes on the same foot were pinched in succession until either a response occurred, or there was an escape attempt. If a nocifensive response occurred, the ipsilateral toes were pinched starting with the first and ending with the fifth digit. As mice are prey species, they are prone to thanatosis (playing dead), and can suppress nocifensive withdrawal as they emerge from anesthesia. As such, a contralateral toe was pinched again following ipsilateral toe trials. An absence of a contralateral nocifensive response was almost always followed within 5 s by escape, and so the test was repeated. The test was also repeated if the mouse regained consciousness while the ipsilateral paw was being assessed. The presence or absence of a response to each ipsilateral toe pinch was recorded. If pinch to a particular toe elicited a response 2 d in a row, recovery was assigned to the first.

For the grasping assay the mouse was suspended upside-down from a wire cage lid and the grasping ability of the hindpaw was assessed and scored. Scores were assigned using the following semi-quantitative metric: undirected paw placement = 0, directed paw placement = 1, occasional grasp = 2, consistent grasp = 3. Both tests were conducted every other day starting on the third postoperative day until the experimental endpoint (day 28).

### Electromyography

Four weeks following injury, mice were anesthetized with urethane (3 g/kg in dH_2_O), and their sciatic nerves were exposed at mid-thigh. The nerves were bathed in paraffin oil and draped across a pair of silver wire hook electrodes (anode-cathode distance: 1 mm). EMG needle electrodes were placed subcutaneously over the lateral aspect of the hindpaw; one at the calcaneus, the other just proximal to the base of the 5th digit (over the abductor digiti minimi muscles). The nerve stimulated with 200 μs square wave current pulses using a stimulus generator (Master 9, A.M.P.I.) and stimulus isolator (A.M.P.I.). Signals were amplified using a Dual Bio-amp connected to a 16 channel Powerlab (ADInstruments). Signals were sampled at 40 kHz and filtered using LabChart7 software. After establishing appropriate electrode polarity, current pulses were delivered at increasing intensities until an EMG signal became apparent, and threshold current was recorded. The maximum EMG signal was then obtained, and the latency and amplitude of the first positive peaks were recorded.

### Tissue processing

**Figure 1.**
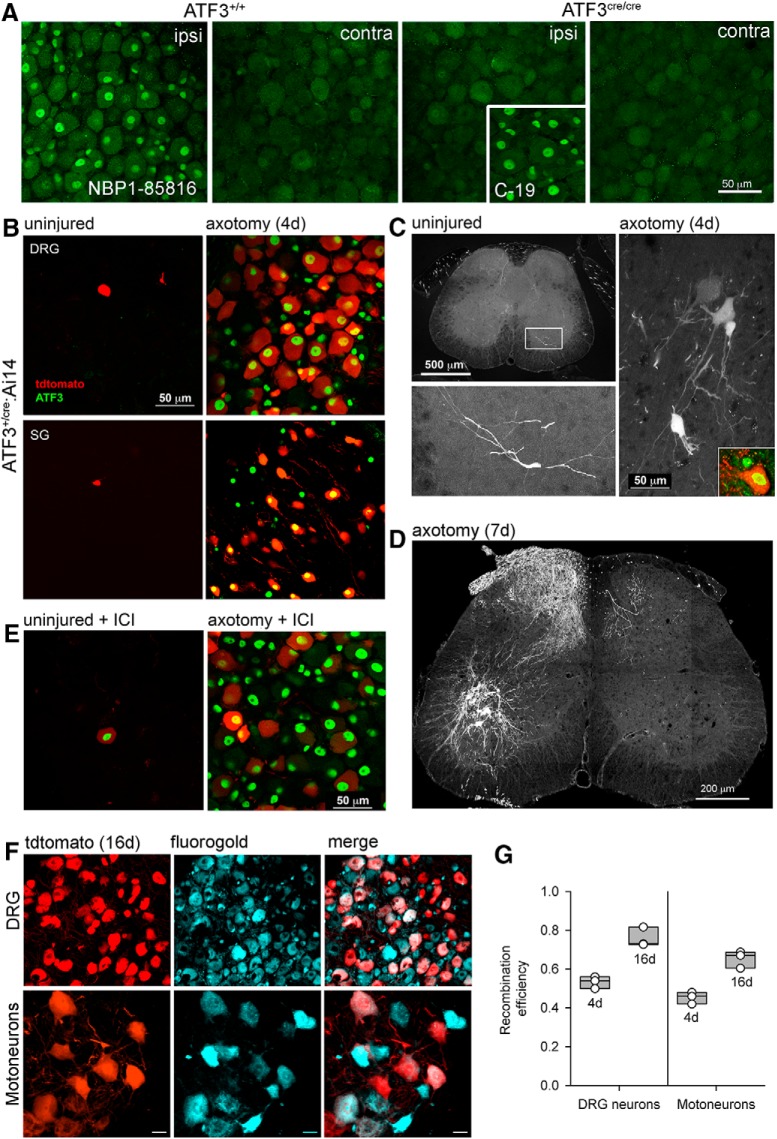
Axotomy-induced recombination in peripherally-projecting neurons. ***A***, Validation of an ATF3-specific antibody. The Novus antibody (NBP1-85816) produces a positive signal in nuclei of axotomized sensory neurons in ATF3^+/+^ mice, but not ATF3^cre/cre^ mice. Note that the Santa Cruz antibody (C-19) labels neuronal nuclei in in the latter (inset), indicating non-specific staining. ***B***, ***C***, Axotomy induced reporter expression in sensory (DRG), sympathetic (stellate ganglion, SG), and motoneurons 4 d after injury. ***D***, Reporter expression in sensory axons and motoneurons one week after injury. ***E***, Preventing CreERT2 translocation from cytoplasm to nucleus with ICI 182780 reduces recombination in ATF3^+^ cells (by ∼50%). ***F***, Recombination efficiency 16 d after injury was calculated by expressing the proportion of tracer-filled somata (labeled at the time of injury) that were also reporter (tdtomato)-positive. ***G***, Recombination efficiencies at 4 and 16 d after injury (*n* = 3 for each time point) for DRG and motoneurons. Images in panels ***A***, ***B***, ***E*** were taken from whole mounts, those in ***C***, ***D***, ***F*** from cryosections.

Mice were transcardially perfused with phosphate buffered saline followed by 4% paraformaldehyde (ThermoFisher). Once dissected, tissue was postfixed overnight in 4% paraformaldehyde, then overnight again in 20% sucrose (ThermoFisher) in 0.1 M phosphate buffer. The tissue was then frozen in Cryomatrix (ThermoFisher) and sectioned at 20 μm (DRG and sciatic nerve) or 50–100 μm (spinal cord). In some cases (Fig. 1), whole sympathetic or sensory ganglia were stained and imaged. All sections were blocked in 10% normal donkey serum with 0.2% Triton X-100 plus 0.02% sodium azide in PBS. Sections were incubated overnight with primary antibodies: rabbit anti-ATF3 (1:400, Santa Cruz SC-188), rabbit anti-ATF3 (1:500, Novus NBP 1-85816), rabbit anti-SCG10 (1:1000, Novus NBP 1-49461), rabbit anti-PTEN (1:400, Cell Signaling 9188), and mouse anti-PTEN (1:200, Cell Signaling 14642). Sections were incubated for 2 h with the appropriate secondary antibodies at a concentration of 1:1000: Alexa Fluor 488 donkey anti-rabbit (Invitrogen A21206), and Alexa Fluor 488 donkey anti-mouse (Jackson ImmunoResearch 715-545-151). Slides were cover-slipped with ProLong Gold with DAPI (Invitrogen).

### Image acquisition and quantification

All images were acquired with a Zeiss LSM 800 confocal microscope using Zen (Blue) software. Recombination efficiency 4 d after lesion was determined by dividing the number of ATF3^+^-plus-tdtomato^+^ neuronal nuclei by the total number of ATF3^+^ neuronal nuclei. The 16-d recombination efficiency was calculated by dividing the number of tracer-positive neuron cell bodies by the total number of reporter-positive cell bodies. All image processing and quantification was done using ImageJ (Fiji version 2.0.0-rc-66/1.52b).

Both PTEN antibodies produced specific staining, but with high background, varying depending on tissue examined (DRG and spinal cord). After comparing both antibodies across all tissues, our analyses relied on the Cell Signaling 9188 antibody for the DRG images and the Cell Signaling 14642 for the ventral root images. PTEN immunoreactivity in the DRG was quantified by measuring the mean pixel intensity of the entire cell layer of the DRG. In the spinal cord, PTEN immunoreactivity was weak in motoneurons, but intense in motor axons on either side of the ventral root exit zones, and so we focused our attention there. PTEN immunoreactivity in ventral root axons was determined by selecting for tdtomato^+^ axons and measuring the PTEN intensity of each axon.

To determine the regeneration density and distance along the sciatic nerve one best 20-μm-thick section (i.e., lacking folds, bubbles or other sectioning artifacts) was selected for each animal, which was then fully imaged (z-stack and tiled). The stack was orthogonally projected into a single image through its entire depth. The images were then processed to generate binary overlays. The average density of SCG10 immunopositive axons along the width of the nerve was measured and then averaged over 100-μm increments.

### Statistics

All statistical analyses were conducted using Prism 7 (GraphPad). We used a one-way ANOVA followed by Tukey’s multiple comparison test to compare PTEN immunoreactivity among DRGs (ipsilateral and contralateral from ATF3-CreERT2 mice with and without floxed PTEN. We used the Kolmogorov–Smirnov goodness-of-fit test to compare PTEN immunoreactivity in tdtomato+ axons between ATF3-CreERT2 tdtomato reporter mice with and without floxed PTEN. Differences in axonal regeneration between ATF3-CreERT2 mice with and without floxed PTEN were determined on cumulative axon densities over 2-mm increments from the crush injury using either a one-way ANOVA followed by Dunnett’s multiple comparison test (three groups) or an un-paired two-tailed *t* test (two groups). For toe pinch, we compared proportions of animals with five sensate digits (i.e., complete nocifensive recovery) over time following injury using a log-rank (Mantel–Cox) test. The same test was used to compare proportions of animals which showed consistent grasping with the injured hindpaw. For EMG data, we compared ipsilateral and contralateral values (threshold, latency, amplitude) within groups using paired *t* tests. Because we used males and females (which differ in size), for between-group comparisons we used unpaired *t* tests on ipsilateral/contralateral ratios.

## Results

### ATF3-driven injury-induced recombination

The Novus ATF3 antibody labeled the injured wild-type mice (ATF3^+/+^) ipsilateral DRG neurons but did not label the same ipsilateral DRG neurons in the homozygous mutant mice (ATF3^cre/cre^; Fig. 1*A*). However, the Santa Cruz antibody did label the injured ipsilateral DRG neurons in the ATF3^cre/cre^ mice, suggesting that it is not specific to ATF3 but another, injury-dependent protein as there was no neuronal labeling of any contralateral DRGs (Fig. 1*A*).

ATF3-driven recombination was exceedingly rare in the uninjured nervous system (Fig. 1*B*,*C*), although it was noted that uninjured recombination appeared to be more prevalent in older mice (data not shown). Four days after PNI, there was a robust tdtomato signal in the neuronal cell bodies in axotomized DRGs, stellate (sympathetic) ganglia (Fig. 1*B*), and ventral motor pools (Fig. 1*C*,*D*) in the ATF3^+/cre^ Ai14 reporter mice without the administration of tamoxifen. Tdtomato expression was only present in neurons, and only in those with nuclear ATF3 immunopositivity (Fig. 1*B*,*C*).

ATF3 expression is maximal at 4 d after injury but declines in sensory and motoneurons between 10 and 20 d (Tsujino et al., 2000). In 4-d lesions, we calculated the proportion of ATF3-positive DRG and motor neurons that were also tdtomato-positive (Fig. 1*B*,*G*). For 16-d lesions, we identified injured neurons by fluorogold labeling (injected at the time of injury), and determined the proportion that were also tdtomato labeled (Fig. 1*F*,*G*). In the DRG, recombination efficiencies were (mean ± SEM) 53 ± 2% (4 d) and 76 ± 3% (16 d). For motoneurons, the efficiencies were 45 ± 2% (4 d) and 65 ± 3% (16 d). When the estrogen receptor α antagontist (ICI) was administered the amount of neuronal recombination was reduced by almost 50% (from 53 ± 2% to 28 ± 1%, *p* < 0.05, *n* = 3 mice per group; Fig. 1*E*), demonstrating that recombination was due to leak of the CreERT2 construct into the nucleus as a result of inadequate cytoplasmic anchoring.

**Figure 2.**
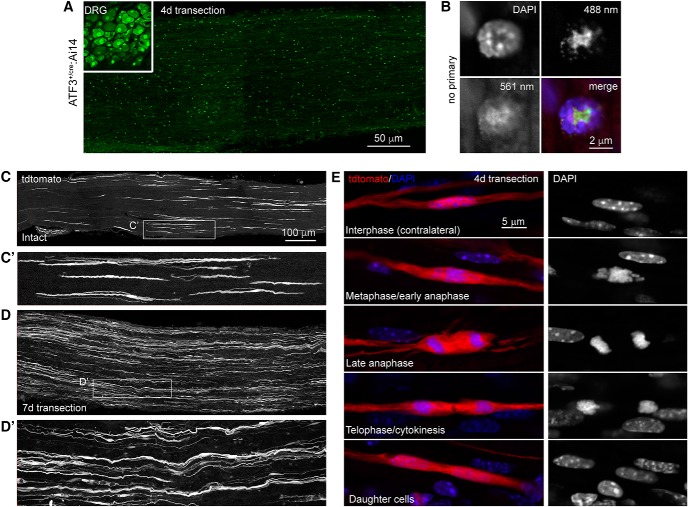
Axotomy does not induce ATF3 in Schwann cells. ***A***, Cryosections from injured DRG (inset) and distal sciatic nerve from the same mouse processed for ATF3 immunohistochemistry (Novus NBP1-85816). ***B***, Punctate staining in the nerve proved to be non-specific fluorescence of leukocytes (note non-nuclear signal in the absence of primary antibody). ***C***, In intact sciatic nerves, cells morphologically identical to Remak cells had at some point undergone recombination. ***D***, Following injury, their numbers increased. ***E***, This was attributable to their proliferation in the injured nerve (as opposed to ATF3 induction and subsequent recombination). ***C′***, ***D′***, Magnification of areas outlined in ***C*** and ***D*** demonstrate the spindle shaped morphology characteristic of Remak cells.

In the injured distal stump of the sciatic nerve, the Novus ATF3 antibody showed a uniform and punctate ATF3 signal that was attributable to white blood cells (Fig. 2*A*). When only the secondary antibody was used the same signal was present and not localized to the nucleus of the putative white blood cells indicating that the antibody signal was an artifact, and that there was no ATF3 upregulation in the injured sciatic nerve (Fig. 2*B*).

In the uninjured sciatic nerve, long, spindle shaped cells that morphologically resemble Remak Schwann cells (RSCs; Gomez-Sanchez et al., 2017) were tdtomato+ and therefore had expressed ATF3 at some point in their lifetime (Fig. 2*C*). Seven days after axotomy, there was an increased density of these tdtomato+ RSCs (Fig. 2*D*), although they were not positive for ATF3 immunolabeling. On closer inspection 4 d after injury, we found multiple examples of tdtomato+ RSCs undergoing all phases of mitosis (Fig. 2*E*), suggesting that the increased density was not due to an injury induced upregulation of ATF3 but instead was the result of proliferation of previously-labeled cells.

### ATF3’s role in peripheral regeneration and functional recovery

Before employing the ATF3^cre^ line to edit the genes of injured neurons to manipulate the regenerative response we first wanted to characterize the effect of losing one or both copies of the *Atf3* allele. Since the Cre insert prematurely terminates the ATF3 coding sequence, the ATF3^cre^ allele is notionally non-functional.

**Figure 3.**
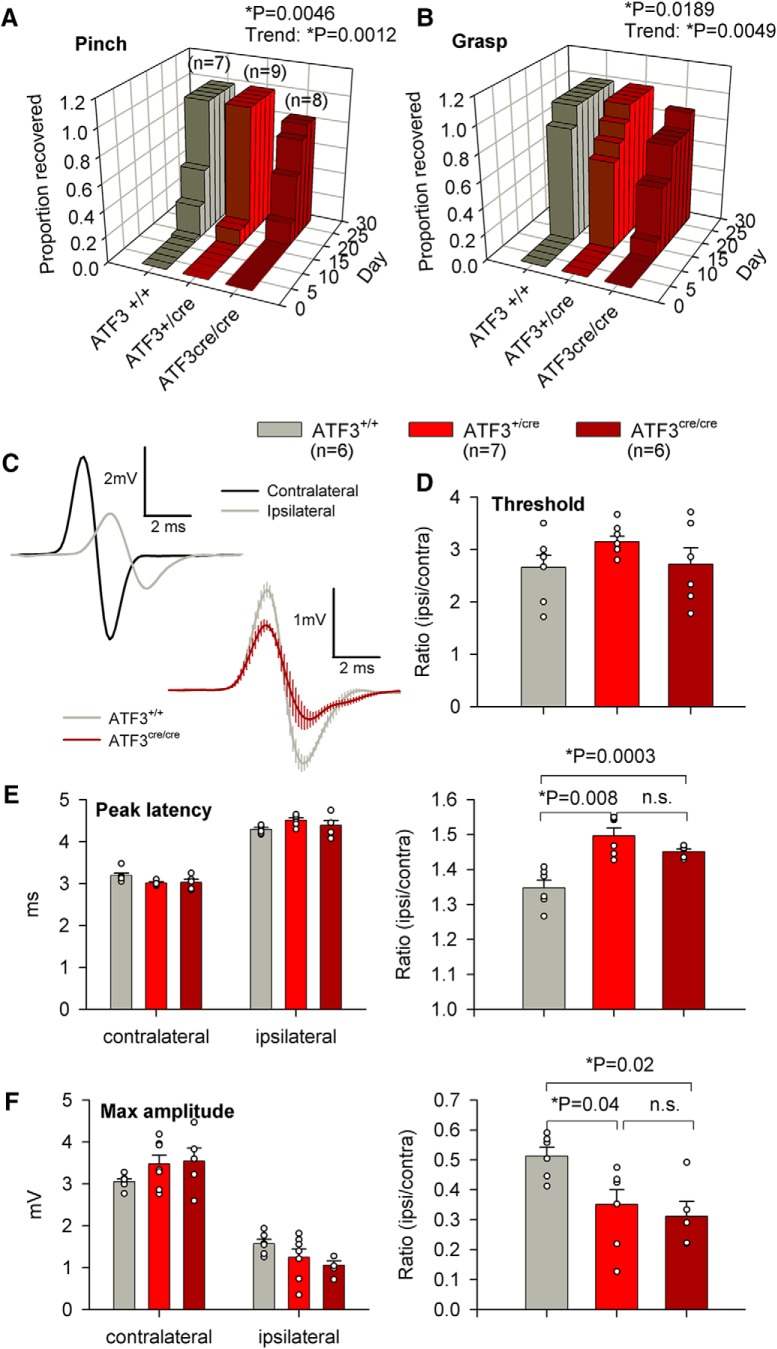
Loss of ATF3 function delays functional recovery following sciatic nerve crush. ***A***, ***B***, The rate of functional recovery (nocifensive reflex withdrawal to a toe pinch and presence of any grasping ability) was reduced in mice lacking both wild-type ATF3 alleles (log rank Mantel–Cox test). Haploinsufficiency was also suggested by the statistically-significant trend from wild-type to homozygous knock-in (log rank Mantel–Cox test), *n* = 7, *n* = 9, and *n* = 8 for ATF3^+/+^, ATF3^+/cre^, and ATF3^cre/cre^, respectively. ***C***, Representative EMG traces from ipsilateral and contralateral sides of an ATF3^+/+^ mouse 28 d after sciatic nerve crush, and composite traces from seven ATF3^+/+^ mice and seven ATF3^cre/cre^ mice. ***D***, EMG thresholds did not differ between genotypes (paired *t* test). ***E***, ***F***, While absolute latencies and amplitudes did not differ between genotypes (paired *t* test), their ipsilateral/contralateral ratios (correcting for mouse size) indicated reduced conduction velocity (***E***) and extent of reinnervation (***F***) in mice lacking one or both wild-type ATF3 alleles (unpaired *t* test), *n* = 6, *n* = 7, and *n* = 6 for ATF3^+/+^, ATF3^+/cre^, and ATF3^cre/cre^, respectively.

To determine whether loss of the *Atf3* allele attenuated functional recovery after PNI we examined behavioral recovery up to 28 d following sciatic crush, and electromyographical (EMG) activity at the experimental endpoint. We found that for both the pinching and grasping assays that the ATF3^cre/cre^ group had significantly diminished functional recovery (*p* = 0.0046 for pinch, *p* = 0.0189 for grasp; Fig. 3*A*,*B*). The performance of ATF^cre/+^ mice was intermediate for both assays (*p* = 0.0012 for pinch, *p* = 0.0049 for grasp) suggesting a gene dosage effect (the amount of functional transcript affects the level of recovery).

In terminal EMG experiments, we found that the ATF3^cre/cre^ group had significantly longer peak latencies (*p* = 0.0003; Fig. 3*E*), and smaller compound muscle action potentials (*p* = 0.002; Fig. 3*F*) when comparing ipsilateral/contralateral ratios to the controls, indicating more complete muscle reinnervation in ATF3^+/+^ mice. There were no differences in activation thresholds (Fig. 3*D*). Surprisingly the ATF^cre/+^ group resembled the ATF^cre/cre^ group in EMG measures, displayed significantly longer peak latencies (*p* = 0.008; Fig. 3*E*), and smaller compound muscle action potentials (*p* = 0.04; Fig. 3*F*) when comparing ipsilateral/contralateral ratios to the controls providing further evidence of a gene dosage effect.

**Figure 4.**
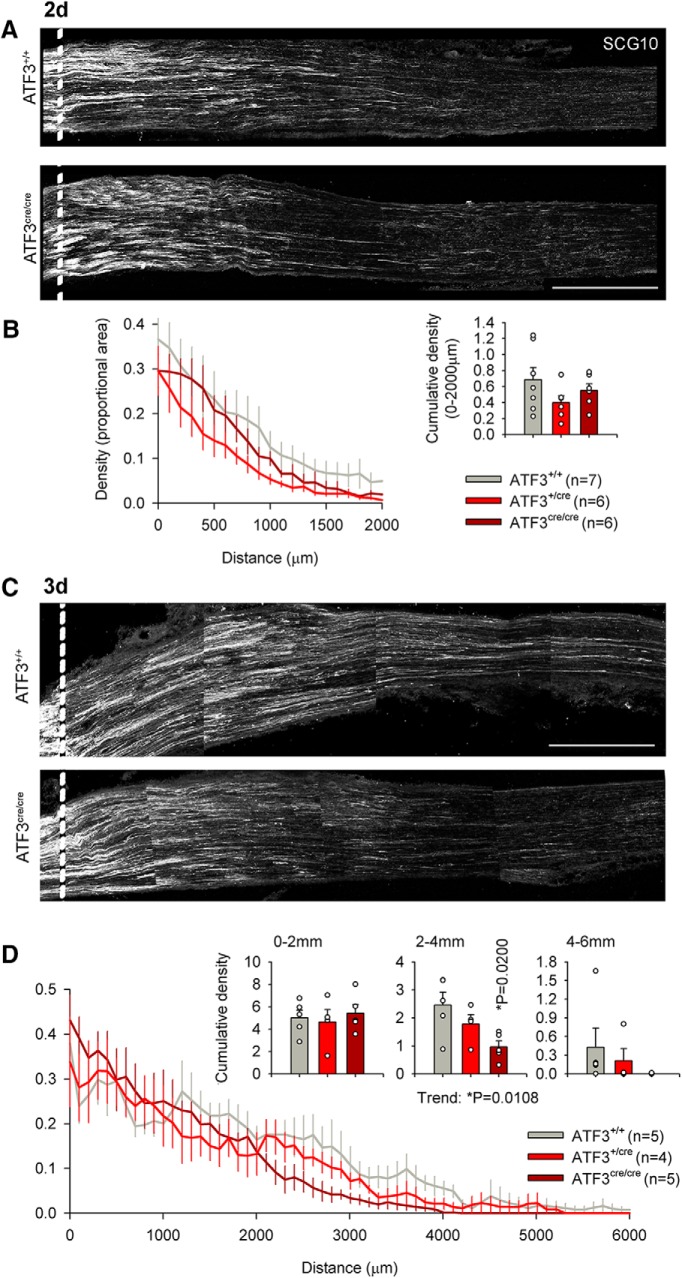
Loss of ATF3 function modestly reduces axonal regeneration following sciatic nerve crush. ***A***, ***B***, There was no difference in axonal regeneration 2 d following injury between ATF3^+/+^, ATF3^+/cre^, and ATF3^cre/cre^ mice (*n* = 7, *n* = 6, and *n* = 6, respectively, groups were compared with a one-way ANOVA on cumulative densities). ***C***, ***D***, ATF3^cre/cre^ mice exhibited significantly diminished axonal regeneration 3 d following injury 2–4 mm distal to the injury compared to ATF3^+/+^ mice (*n* = 5 for both groups, one-way ANOVA followed by Dunnett’s multiple comparison test). The hemizygous group (*n* = 4) tested positive as a significant intermediary between both control and ATF3 null groups (*post hoc* test for trend). Dotted line indicates distal border of crush site, 500 μm from the edge of the block. Scale bars: 500 μm.

The above data unequivocally demonstrate delayed functional recovery (which can only be attributable to regeneration of injured axons given the absence of ATF3 expression in uninjured neurons) in ATF3-deficient mice. We then asked when differences in regeneration could be discerned between genotypes anatomically. To this end we assayed axonal regeneration along the sciatic nerve 2 and 3 d after crush by taking a single section (one that lacked sectioning artifacts like tears or folds) and imaged through its full 20-μm depth) for analysis. Two days after sciatic nerve crush, cumulative axon density was not yet different between ATF3^+/+^, ATF3^cre/+^, and ATF3^cre/cre^ groups (Fig. 4*A*,*B*). Three days after sciatic nerve crush, a significant difference was detected in cumulative axonal density at 2–4 mm distal to the injury site between ATF3^+/+^ and ATF3^cre/cre^ groups (*p* = 0.0200), and the ATF3^+/cre^ group tested positive as a significant intermediary between groups (*p* = 0.0108; Fig. 4*C*,*D*). These anatomic results further support our findings that loss of one or both copies of the ATF3 allele diminishes regeneration after PNI.

### ATF3-driven injury-induced PTEN knock-down

**Figure 5.**
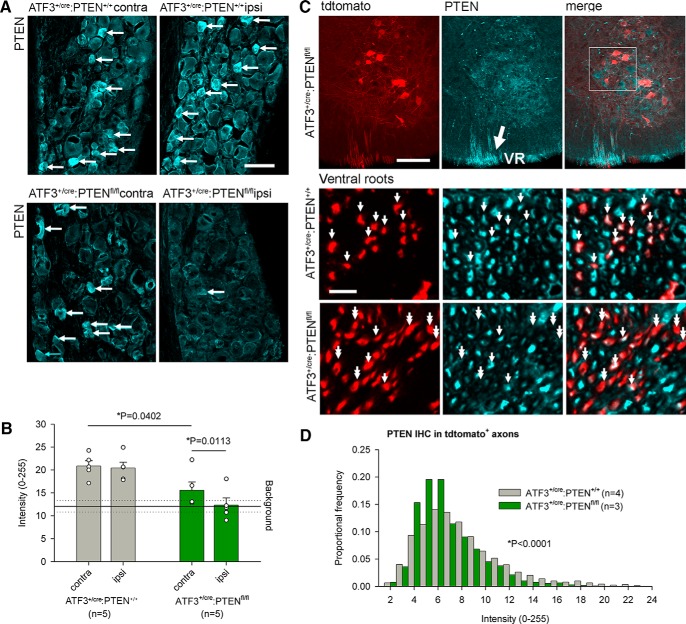
Axotomy-induced PTEN deletion in sensory and motoneurons. ***A***, ***B***, Axotomy results in significant loss of PTEN expression in the DRG. Arrows in ***A*** indicate small PTEN-positive DRG neurons. The PTEN antibody results in high background staining in all animals, to which PTEN immunoreactivity is reduced in axotomized DRGs of ATF3^+/cre^:PTEN^fl/fl^ mice (***B***; one-way ANOVA followed by Dunnett’s multiple comparison test, *n* = 5 for both groups). ***C***, PTEN immunoreactivity is weak in all motoneurons, rendering difficult confirmation of axotomy-induced knock-down. However, ventral root (VR; large arrow) axons close to the ventral root exit zone were intensely immunopositive, single arrows indicate axons that were both tdtomato and PTEN positive, whereas double arrows indicate recombination without PTEN immunoreactivity. In sections of ventral roots, we were able to demonstrate a significant decrease in PTEN immunoreactivity in tdtomato-positive axons (***D***; Kolmogorov–Smirnov goodness of fit test), *n* = 4 and *n* = 3 for ATF3^+/cre^:PTEN^+/+^ and ATF3^+/cre^:PTEN^fl/fl^, respectively. Scale bars: 50 μm (***A***), 100 μm (***C***), 10 μm (***E***).

To determine whether the ATF3^+/cre^ line can excise floxed endogenous genes, we crossed it to a floxed *Pten* line, crushed the sciatic nerve, and measured PTEN expression 28 d after injury. Three sections each from five animals were used for analysis. High background with both PTEN antibodies rendered precise estimates of recombination efficiency difficult, but the antibody labeled small-diameter DRG neurons, as reported previously by Gallaher and Steward (2018). There was no reduction in PTEN immunoreactivity between the ipsilateral and contralateral DRG cell layers in control animals (Fig. 5*A*) indicating that injury itself does not change PTEN expression. For reasons unknown, but possibly due to the leaky Cre-ERT2 construct, there was a significant difference in intensity measurements between genotypes on the contralateral (uninjured) side. Nevertheless, there was a significant reduction in PTEN expression on the ipsilateral side compared to the contralateral side of ATF3^+/cre^ PTEN^fl/fl^ mice (*p* = 0.0113) indicating that ATF3 driven Cre expression successfully excises the PTEN gene (Fig. 5*A*,*B*). The non-specific background PTEN antibody intensity was determined from sections from ATF3^+/cre^ PTEN^+/+^ mice in which PTEN-positive DRG neuronal somata were excluded from regions of interest (i.e., the ROIs were the negative of the PTEN-positive neurons). PTEN immunoreactivity in ATF3^+/cre^ PTEN^fl/fl^ mice was no different from background.

Spinal motoneurons were weakly PTEN-immunoreactive, and although somata were less readily identifiable if they were also tdtomato-positive (Fig. 5*C*), background staining precluded reliable analysis in the ventral horn. We therefore examined PTEN expression in the ipsilateral ventral roots (which were intensely PTEN-immunoreactive on either side of the ventral root exit zone) 7 d after sciatic nerve crush in reporter mice with ATF3^+/cre^ and PTEN^fl/fl^ or PTEN^+/+^ alleles. Four control animals and three experimental animals were used (three sections from each). We found that PTEN expression was significantly reduced (*p* < 0.0001) in the axons that also expressed tdtomato in the PTEN^fl/fl^ group compared to controls (Fig. 5*D*).

### ATF3-driven PTEN excision improved functional recovery

**Figure 6.**
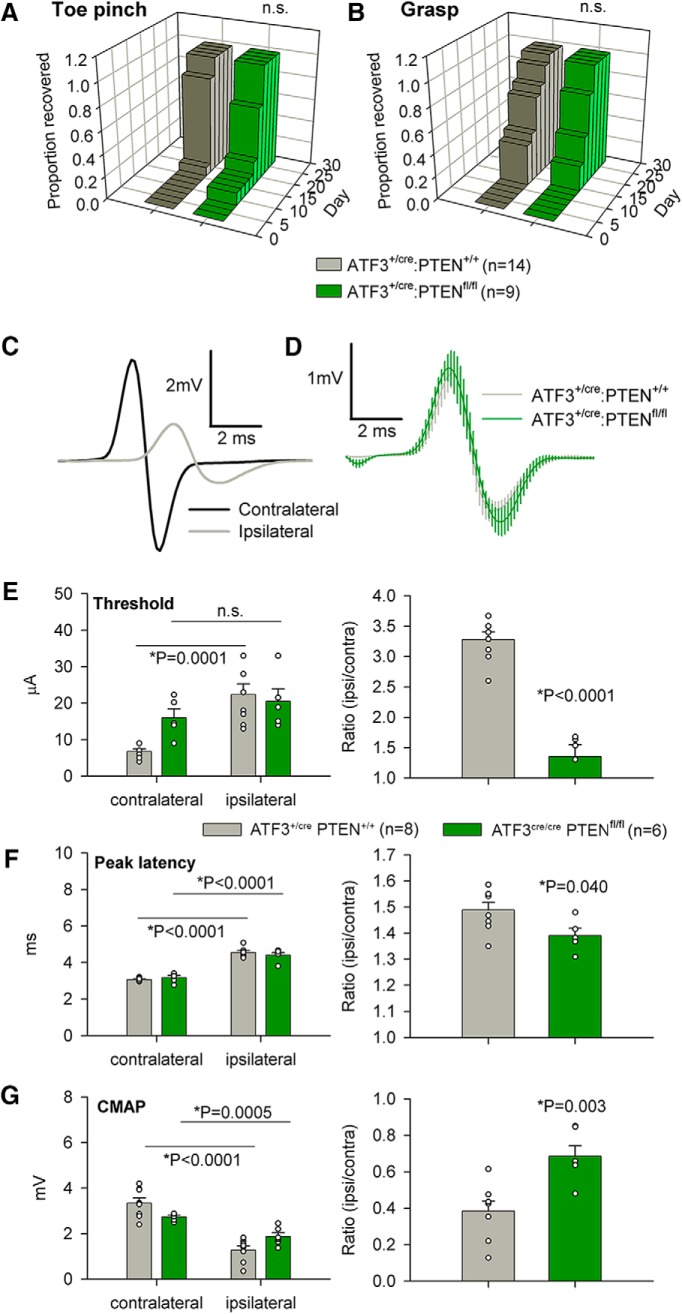
Axotomy-induced PTEN deletion and improves functional recovery following sciatic nerve crush. ***A***, ***B***, While there was no difference in recovery of reflex nociception or grasping in mice with floxed PTEN alleles (log rank Mantel–Cox test, *n* = 14 and *n* = 9 for ATF3^+/cre^:PTEN^+/+^ and ATF3^+/cre^:PTEN^fl/fl^, respectively), EMG responses (***C–G***) indicated enhanced recovery of neuromuscular function. ***C***, ***D***, Representative EMG traces from ipsilateral and contralateral sides of an ATF3^+/cre^:PTEN^+/+^ mouse 28 d after sciatic nerve crush (***C***) and composite traces from eight ATF3^+/cre^:PTEN^+/+^ mice and six ATF3^cre/cre^:PTEN^fl/fl^ mice. ***E–G***, Ipsilateral/contralateral ratios of EMG thresholds (***E***), peak EMG latencies (***F***), and maximum CMAP amplitudes (***G***) all indicated more complete muscle reinnervation 28 d after injury; *n* = 8 and *n* = 6 for ATF3^+/cre^:PTEN^+/+^ and ATF3^+/cre^:PTEN^fl/fl^, respectively, averages were compared using paired *t* tests and ipsilateral/contralateral ratios with unpaired *t* tests.

To determine whether the ATF3^+/cre^ driven *Pten* excision was robust enough to effect enhanced functional recovery after PNI we examined behavioral recovery over a month following sciatic crush, and EMG activity at the experimental endpoint. For behavioral analysis the control group had 14 animals in it and the experimental group had nine. There were no differences in sensory (pinch) or sensorimotor (grasping) assays between the ATF3^+/cre^ PTEN^fl/fl^ and control groups (Fig. 6*A*,*B*), as has been reported previously (Gallaher and Steward, 2018).

In terminal EMG experiments, however, we found that the ATF3^+/cre^ PTEN^fl/fl^ group had a significantly lower EMG activation threshold (*p* < 0.0001; Fig. 6*E*), shorter peak latencies (*p* = 0.040; Fig. 6*F*), and larger compound muscle action potentials (*p* = 0.003; Fig. 6*G*) when comparing ipsilateral/contralateral ratios to the controls, indicating more complete muscle reinnervation in mice lacking PTEN.

**Figure 7.**
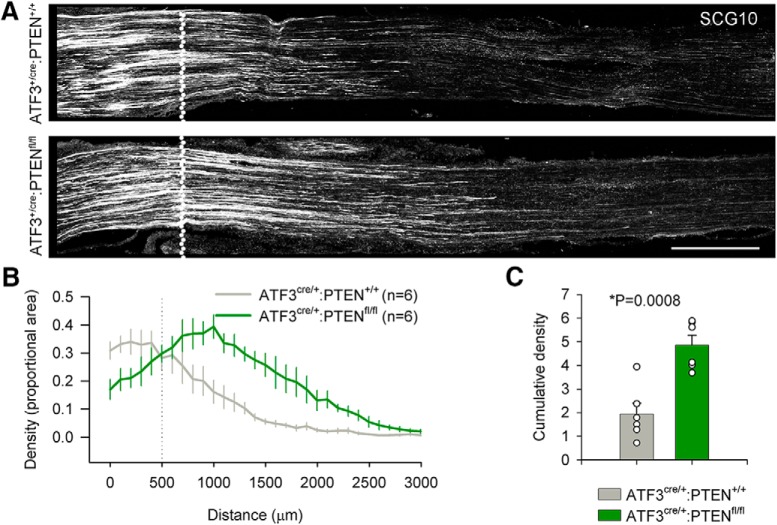
Axotomy-induced PTEN deletion and anatomic regeneration following sciatic nerve crush. ***A***, ***B***, Axonal regeneration 2 d subsequent to sciatic nerve crush (dotted line) was significantly enhanced in ATF3^+/cre^:PTEN^fl/fl^ mice. In ***C***, bar graphs represent the cumulative density of SCG10 immunoreactivity from 0 μm (the distal extent of the crush site) to 2000 μm; *n* = 6 for both groups and cumulative densities were compared using an unpaired *t* test. Scale bars: 500 μm.

Enhanced functional recovery in PTEN deficient mice implies more robust axonal regeneration. We were again curious as to when axonal regeneration following ATF3-driven injury induced *Pten* excision might be detected histologically following sciatic crush injury. Six animals were used in each group and a single section (lacking sectioning artifacts, and imaged through its full 20-μm depth) was taken for analysis. Two days after sciatic nerve crush, axon density plotted as a function of distance was obviously increased in ATF3^+/cre^ PTEN^fl/fl^ over that in ATF3^+/cre^ PTEN^+/+^ mice, and the cumulative density at 2 mm was statistically greater (*p* = 0.0008; Fig. 7). This demonstrates that the ATF3^+/cre^ line is able to edit the genes of injured neurons with enough efficacy to produce anatomic differences in regeneration.

### ATF3-driven recombination after CNS injury

**Figure 8.**
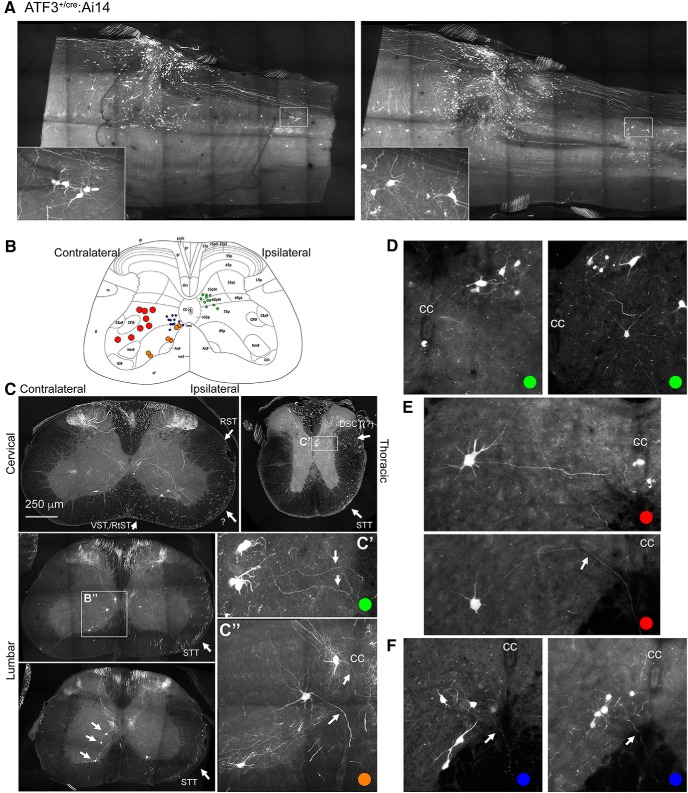
Axotomy-induced recombination one week following spinal hemisection: spinal cord. ***A***, Two examples of the injury site from separate animals in longitudinal section. Insets show consistently-recombining ipsilaterally-projecting neurons near Clarke’s column below the injury. ***B***, Cartoon of transverse section of the hemisected spinal cord illustrating relative positions of positionally and morphologically distinguishable recombined neurons (colored dots correspond to examples in ***C–F***). ***C***, Examples of recombination after injury in cervical (top left), lumbar (two examples middle and bottom-left), and thoracic (top right). The most consistent findings were small ipsilaterally-projecting neurons in the thoracic cord (***C’***), and large neurons contralateral to injury from just lateral to area X to the ventral gray matter in the lumbar cord (***C’’***). Arrows in ***C’***, ***C’’*** indicate midline-crossing axons. Arrow pointing to tdtomato^+^ axons in dorsal cervical white matter indicates probable rubrospinal (RST) and/or raphespinal tracts. Arrows pointing to reporter-positive axons in ventral white matter indicate probable reticulospinal (RtST) and vestibulospinal (VST), and an unknown descending projection (?) tracts rostral to the injury, and spinothalamic (STT) and possible dorsal spinocerebellar [DSCT(?)] below the injury (thoracic and lumbar sections). ***D***, Neurons in Clarke’s column ipsilateral to the hemisection. ***E***, ***F***, Large and small, respectively, putative spinothalamic tract neurons contralateral to the hemisection. Arrows in ***E***, ***F*** indicate commissural axons; cc, central canal.

**Figure 9.**
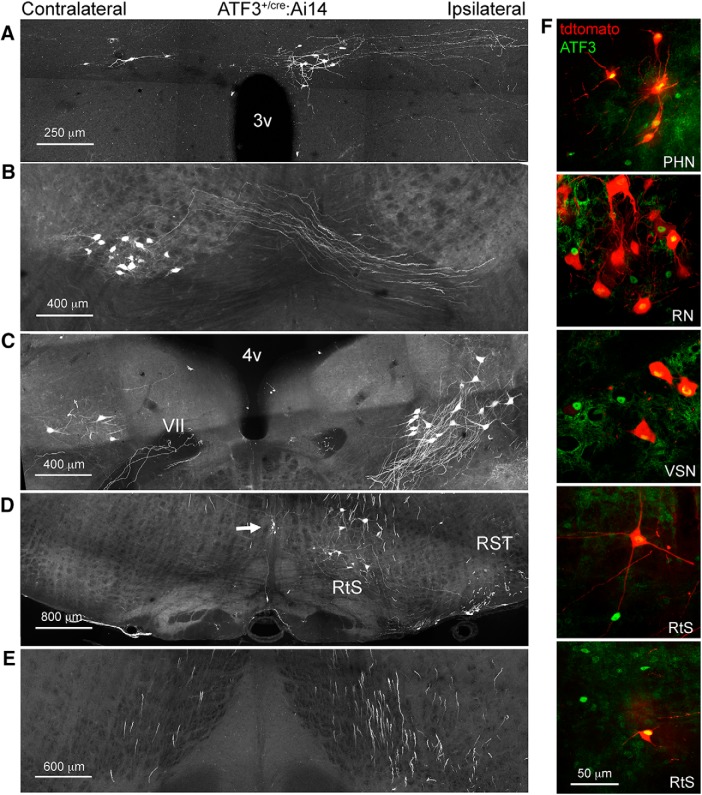
Hemisection-induced recombination in supraspinal neurons one week after injury. ***A***, Paraventricular hypothalamic nucleus, descending part. ***B***, Red nucleus. ***C***, Vestibulospinal nucleus (the genu of the facial nerve is indicated by VII). ***D***, Reticulospinal neurons (RtS) and raphespinal neurons (arrow), and the rubrospinal tract (RST). ***E***, Reporter-expressing axons in the medial longitudinal fasciculus (conveying descending projections of reticulospinal and vestibulospinal axons). ***F***, Examples of ATF3-positive, tdtomato-positive and tdtomato-negative neurons in the paraventricular hypothalamic nucleus (PHN), red nucleus (RN), vestibulospinal nucleus (VSN), and reticulospinal neurons (RtS).

After confirming that the ATF3^cre^ line is capable of efficient recombination that can selectively edit genes the genes of injured neurons after PNI we wanted to determine whether the same line was applicable to CNS injury. Six ATF3^cre/+^:Ai14 reporter mice underwent a C8/T1 lateral spinal hemisection, were killed 7 d after injury, and recombination throughout the entire CNS was characterized. In the spinal cord, several tracts were reliably labeled, these included putative rubrospinal, raphespinal, reticulospinal, and vestibulospinal descending tracts (Fig. 8*C*,*D*). Additionally, ascending neurons were tdtomato+; based on anatomic position these are likely to be spinothalamic spinocerebellar neurons (Fig. 8*E*,*F*). In the cervical cord, rostral to the injury site, there was a substantial amount of recombination in primary afferents innervating the dorsal horn (Fig. 8*C*). Four deep brain nuclei were consistently reporter-positive, these were the rubrospinal, reticulospinal, vestibulospinal, and paraventricular hypothalamic nuclei (Fig. 9*A–E*). There were examples of ATF3 immunolabeled and tdtomato-positive neurons in each of these nuclei (Fig. 9*F*).

## Discussion

The expression of Cre recombinase has for the most part been restricted to either developmentally distinct subpopulations of cells via its insertion under specific promoters or to distinct physical regions by viral transfection. Selective cellular genetic modification of neurons based on functional state is an attractive and important refinement to this approach. Guenthner et al. (2013), for example, used a genetic labeling technique controlled by neuronal activity. In this case, recombination was driven by promoters for the immediate-early genes Fos and Arc, upregulated as part of the “excitation-transcription” neuronal response to synaptic activity initiated by CREB phosphorylation. Here, we present a novel transgenic mouse line that expresses Cre only once peripherally-projecting neurons have been axotomized by inserting its construct into the native ATF3 locus. We demonstrate a substantial amount of recombination (∼50% by 4 d after injury, rising to ∼65–80% by 16 d) in injured sensory, motor, and sympathetic neurons that is selective to axotomy. This injury-dependent Cre expression is restricted to the neurons that have been axotomized, is rare in uninjured controls, and absent in peripheral glial cells. Furthermore, we show that axotomy-induced Cre expression can excise floxed genes with sufficient efficacy to significantly effect anatomic regeneration and functional recovery.

It is important to note that despite the Cre recombinase being anchored to a mutated estrogen receptor (ERT2) we have achieved significant recombination without the administration of tamoxifen. This can be attributed to the “leakiness” of the ERT2 construct where the ERT2 protein overwhelms its cytoplasmic anchor and translocates to the nucleus without tamoxifen (or its metabolites) binding. The high degree of tamoxifen-independent recombination we report is likely driven by massive upregulation of ATF3 in the PNS after injury: the *Atf3* gene (and hence Cre-ERT2) is highly transcribed once the neuron is axotomized, and there is a greater likelihood of ERT2 leak and subsequent recombination. Reduction of recombination by ICI 182780 (Fig. 1) provides an opportunity to titrate recombination due to leakiness.

We did not use tamoxifen to improve the recombination efficiency of the model, as it has been previously reported that tamoxifen administration, at the standard dose of 75 mg/kg, upregulates ATF3 without injury (Denk et al., 2015). Notionally it is possible that a lower dose of tamoxifen would not induce ATF3 expression, but would still improve the recombination efficiency after injury, although this dose has yet to be determined. Regardless, the efficiencies achieved in this report due to ERT2 leak remain sufficient to excise *Pten* and significantly improve anatomic regeneration and functional recovery.

The potential utility of our model is strengthened by similarities to previous manipulations using different techniques. A study by Gallaher and Steward (2018) investigated the effect of *Pten* deletion following axotomy in the sensory neurons that innervate the sciatic nerve. They excised *Pten* through a more traditional intraganglionic injection of an AAV Cre vector into the L4 and L5 DRG. Their study and ours agree on three key results: (1) PTEN immunohistochemistry preferentially labels small diameter sensory neurons, (2) PTEN deletion increased axonal regeneration along the sciatic nerve 3 d after axotomy, and (3) PTEN deletion after sciatic nerve crush did not significantly improve sensory functional recovery. The ATF3-CreERT2 line has several distinct advantages over more traditional Cre delivery models; the technical viral transfection setup is not necessary and Cre can be expressed in neuronal populations that may not be amenable to local injection. Moreover, the ATF3-ERT2 line allows for the selective editing of only injured neurons where a viral Cre transfection does not preclude the possibility of Cre expression in uninjured neurons.

Using a homozygous knock-in (i.e., null mutant), we tested the validity of two reported ATF3-specific polyclonal antibodies: Novus (NBP 1-85816) and Santa Cruz (C-19). ATF3 signal was expectedly absent in the ATF3 null ipsilateral DRG after axotomy when using the Novus antibody. However, signal was still present in both the control and ATF3 null ipsilateral (but not contralateral) DRG when tested with the Santa Cruz antibody suggesting that is not specific to ATF3, but likely another injury-dependent protein. This could call into question some of the conclusions made by the over 150 articles that have used this antibody. We then used the Novus ATF3 antibody to investigate ATF3 expression in the injured sciatic nerve. Despite the appearance of putative ATF3 upregulation, we were able to determine that this signal was restricted to white blood cells, non-nuclear, and in fact due to background fluorescence. Clements et al. (2017) reports Schwann cell ATF3 mRNA expression in the uninjured sciatic nerve which is then reduced on injury, which appears to contradict our observations of a lack of ATF3-driven recombination in normal Schwann cells but supports our finding of a lack of injury-dependent glial cell ATF3 expression. To obtain these data the Schwann cells needed to be dissociated, purified, and then sorted before sequencing. Given ATF3’s role as a stress response immediate early gene it is certainly plausible that this baseline ATF3 expression is due to the FACS process and not in fact expressed in the *in vitro* uninjured nerve, this has indeed proven to be an issue in other cell types, such as muscle (van den Brink et al., 2017). Histologic evidence of ATF3 mRNA upregulation following nerve injury is also unconvincing (Hunt et al., 2004). We therefore conclude that ATF3 is not upregulated in peripheral glia after injury cells, contrary to previous reports. This finding further strengthens the usefulness of the model as any axotomy-dependent Cre upregulation is restricted to neuronal populations.

Tdtomato-positive RSCs increased in density following sciatic nerve injury. RSCs are a subtype of Schwann cell that are non-myelinating but still ensheathe small caliber axons to form Remak bundles (Harty and Monk, 2017). Gomez-Sanchez et al. (2017) characterized this subtype of Schwann cells using sporadic permanent fluorophore labeling. These cells are spindle shaped, can be branched, and are ∼250 μm in length; all of which are characteristics of the sciatic nerve cells found to undergo injury-independent recombination in the ATF3^cre^ Ai14 reporter line. The absence of ATF3 immunoreactivity in these cells, along with the high numbers of RSCs that can be observed undergoing mitosis 4 d after injury show that their increase in density is due to cellular division rather than injury-induced ATF3-cre-mediated recombination.

The ATF3^cre/cre^ null mutant also allowed us to test the effect of a loss of ATF3 function in PNS regeneration early after injury. While we found no significant differences between the groups in axonal regeneration along the sciatic nerve at the 2-d time point, we did find a statistically significant functional deficit of both ATF3-deficient groups in both our behavioral and EMG assays. Despite being a standard time point for this type of analysis, the lack of significantly different 2-d axonal regeneration along the sciatic nerve could be because the axons have not had sufficient time to differentially regenerate enough to produce a measurable difference. This supports previous work done by Gey et al. (2016), who found attenuated regeneration after facial nerve axotomy in ATF3 null mice. Gey et al. (2016) also showed that when DRG neurons were cultured in the presence of NGF, any differences in outgrowth between wild-type and knock-out neurons ATF3 were abolished. NGF is produced early on in the distal transected nerve (Heumann et al., 1987), and this early abundance may compensate for any ATF3-mediated regenerative differences. By 3 d after injury, however, a clear difference between ATF3^+/+^ and ATF3^cre/cre^ mice had emerged, along with evidence again for haploinsufficiency (the intermediate position of ATF3^+/cre^ mice). To our knowledge, this is the first evidence that a loss of ATF3 mitigates functional recovery after injury.

Interestingly the existing literature suggests that ATF3 heterozygotes are haplosufficient (Gey et al., 2016) which is contrary to our findings of a gene dosage effect. The fact that the amount of ATF3 transcript affects the functional outcome after peripheral axotomy might seem surprising given how drastically ATF3 is upregulated in neurons after injury. However, given that ATF3 is a bZIP transcription factor that dimerizes with itself or other bZIP transcription factors to affect transcription, it is conceptually possible that the binding affinity for the ATF3 dimerization pair responsible for improving regeneration is low and therefore many copies of ATF3 must be produced for its pro-regenerative effect. Recent work done be Rodríguez-Martínez et al. (2017) exemplified this by showing that ATF3’s DNA binding site preference to be highly dependent on its bZIP dimerization.

ATF3 gene dosage effects (i.e., partial function in hemizygous mice) should not be an impediment to investigations of functions of other (floxed) genes or sequences provided the appropriate controls are used. There is simply a new baseline on which other genetic manipulations (using ATF3-Cre-driven excision of floxed gene “X”) can be evaluated. For this reason it is important that any investigation of the function of gene X compares ATF3^+/cre^:X^+/+^ with ATF3^+/cre^:X^fl/fl^ (or ATF3^+/cre^:X^+/fl^, for investigating possible haploinsufficiency of X), as we have done here substituting “X” with “PTEN.”

ATF3 is not only upregulated after PNI but also after CNS trauma including spinal cord injury (Huang et al., 2007), albeit usually meagerly and/or transiently, rendering expression patterns difficult to reveal (for review, see Hunt et al., 2012). As such we wanted to determine whether CNS neurons could be accessed genetically via ATF3-cre-mediated recombination after a lateral spinal cord hemisection. We observed consistent recombination in four deep brain nuclei (rubrospinal, reticulospinal, vestibulospinal, and hypothalamic) and their projecting axons and in at least two putative ascending tracts (spinocerebellar and spinothalamic). There was no recombination observed in the injured corticospinal tract, but this is not surprising given that it takes intracortical axotomy to induce ATF3 in corticospinal neurons (Mason et al., 2003): the magnitude of upregulation of regeneration associated genes (of which ATF3 is) have been documented to be dependent on the distance from the axon transection to the soma (Fernandes et al., 1999).

The mammalian PNS is able to regenerate after injury, unlike the CNS where regeneration does not occur. This positions the PNS as an excellent model for study to better understand what is necessary for mammalian neuronal regeneration. The ability to edit the genes of injured neurons and further dissect what is necessary and/or sufficient to produce this regeneration is of obvious value, and is now possible with the ATF3^cre^ transgenic line. ATF3 is also expressed in injured neurons after spinal cord injury (Huang et al., 2007; Wang et al., 2015; Darlot et al., 2017), traumatic brain injury (Förstner et al., 2018), and ischemic stroke (Song et al., 2011), making the line useful to scientists interested in the neuronal response to each of those pathologies.

## Synthesis

Reviewing Editor: Mike Fainzilber, Weizmann Institute of Science Decisions are customarily a result of the Reviewing Editor and the peer reviewers coming together and discussing their recommendations until a consensus is reached. When revisions are invited, a fact-based synthesis statement explaining their decision and outlining what is needed to prepare a revision will be listed below. The following reviewer(s) agreed to reveal their identity: Simone Di Giovanni.

This paper reports a potentially useful procedure for labeling injured neurons based on tethering upregulated expression of the ATF3 gene to a reporter protein via the Cre-LoxP system. As the authors point out, ATF3 expression can increase in injured neurons by over 2 orders of magnitude. The authors use an ATF3-CreERT-reporter combination that exhibits very little neuronal expression in uninjured mice. Due to the high induction of AFT3 after injury (and the very high sensitivity of tdT to low levels of Cre), reporter expression is detectable in the absence of tamoxifen due to 'leakiness' of the construct. The authors test this system in PNS and CNS and provide a variety of observations. The bulk of the work seems technically well conducted and the data look of good quality. However, since the authors are proposing this as a technique that has broad potential applications, there are serious concerns regarding limitations of the model that must be clarified and stated clearly for the reader. This and additional issues are detailed below.

1) The authors gloss over a critical point. The model presented uses a 'knockin' of the reporter transgene. Such knockin models render the inserted gene dysfunctional. Heterozygous animals have only one functional gene, and homozygous animals represent full gene deletions or knockouts. In many cases lacking one allele does not matter, in others, such as here, it does.

Such knockin models have advantages over more conventional transgenic insertion models for certain types of studies. However, in the case of generating a model to label injured neurons as presented here, this particular approach is sub-optimal. A conventional transgene insertion model would clearly be more useful as a labeling technique that did not render the labeled cell as being a +/- heterozygote. The AFT3 gene is markedly upregulated and is clearly a critical part of the injury response. The authors' own findings show that the heterozygous version of their knockin-in gene exhibits a haploinsufficiency response in injured neurons. This is particularly important because the knockin gene is a transcription factor, and for this reason, this technique would of limited value for conducting gene-expression analyses. The transgenic labeling procedure proposed here clearly has a pronounced effect on the regulation of the injury response in the labeled neurons, which would render their response abnormal as regards downstream genetic regulation. Thus, any experiments conducted to study genetic responses in these neurons would not be normal, and the responses to any modulatory interventions could not necessarily be viewed as normal. The authors do not mention any of this. Instead, the authors, make strong claims about the usefulness of the procedure in many contexts including to do gene expression analysis of injured neurons. This is not appropriate. The authors should remove the claims regarding gene expression analyses and functional relevance in the abstract and significance summaries. The technique is primarily useful for anatomical labelling, but far less so for genetic or functional studies. The authors should explicitly point this out so as not to mislead potential users of the technique. The authors should clearly address these points in the discussion and discuss the various ways in which AFT3 haploinsufficiency could alter data interpretation.

2) The data presented in the Figures are overall convincing although the authors should report statistical tests used and N in each Figure as presently this makes accurate data interpretation a little limited.

3) A major limitation of the study is the lack of axonal regeneration studies at later time points past day 2 post-injury. While the authors show reduction of functional recovery after ATF3 deletion, they do not study axonal regeneration past day 2, where they did not observe any difference. How can the authors explain this finding if they do not assess regeneration at later time points? Also, in the case of PTEN deletion where they find enhanced functional recovery, they do not study axonal regeneration past day 2. Additional time points measuring axonal regeneration are needed, or removal of claims regarding utility of this system for functional studies of nerve regeneration, as detailed in point 1 above.

4) If the authors wish to argue that their ATF3-related expression patterns in regenerating/sprouting and non-regenerating cell bodies are linked to regenerative ability, they must show expression of a panel of regeneration associate genes in some of the neuronal populations investigated.

## Author Response

We thank the reviewers for their constructive comments and suggestions. We have made major revisions accordingly, which we feel has improved the paper considerably.

1a. The authors gloss over a critical point. The model presented uses a 'knockin' of the reporter transgene. Such knockin models render the inserted gene dysfunctional. Heterozygous animals have only one functional gene, and homozygous animals represent full gene deletions or knockouts. In many cases lacking one allele does not matter, in others, such as here, it does.

Response: The reviewer's principal misgivings in the first point (which we have broken down for ease of responding) arise from a lack of clarity on our part regarding what the appropriate controls are when using these mice. The adjustments to the manuscript and our explanation below should quell any concerns.

It is important to note that reporter is not knocked-in - creERT2 is.

The point of heterozygosity has been given more emphasis. The reviewer is correct in that there is a partial loss of ATF3 function when only one copy of ATF3 is present. Any studies using the mice to edit the genome (via excision of floxed sequences) must use ATF3+/cre mice as controls. We have clarified this point in the methods and discussion. The heterozygosity only matters if heterozygotes are compared to wild-type animals (with respect to ATF3) in studies using these mice for axotomy-induced excision of sequence “X” (i.e. ATF3+/+:Xfl/fl vs ATF3+/cre:Xfl/fl) - this would indeed be inappropriate. We demonstrate this directly in Figure 5A of the revised version: there is a difference between background PTEN immunoreactivity between ATF3+/+ mice and ATF3+/cre mice (both PTENfl/fl). Of course the relevant comparison in terms of axotomy-induced recombination is between axotomized and uninjured neurons on the same background. A new paragraph has been inserted into the discussion to emphasize this point (see response to 1c, below).

1b. Such knockin models have advantages over more conventional transgenic insertion models for certain types of studies. However, in the case of generating a model to label injured neurons as presented here, this particular approach is sub-optimal. A conventional transgene insertion model would clearly be more useful as a labeling technique that did not render the labeled cell as being a +/- heterozygote.

Response: Even for labelling, our knock-in approach has advantages over inserting Cre into ROSA26 or driving it from a BAC. Chiefly, it will ensure that Cre expression faithfully mirrors the intensity and location of Atf3 expression. With a BAC, which usually exists in concatemeric form, modelling endogenous expression levels can be particularly problematic. In the mice we use, the Cre construct is driven by the native ATF3 promoter, in its native locus. When the ATF3 promoter is active, Cre is expressed and (when crossed with floxed-stop reporter mice) tdtomato is expressed as we show in Fig. 1.

1c. The AFT3 gene is markedly upregulated and is clearly a critical part of the injury response. The authors' own findings show that the heterozygous version of their knockin-in gene exhibits a haploinsufficiency response in injured neurons. This is particularly important because the knockin gene is a transcription factor, and for this reason, this technique would of limited value for conducting gene-expression analyses.

Response: Yes, the partial loss of ATF3 function does indeed change the baseline from that of wild-type animals. However, this is simply a new baseline upon which other genetic manipulations (using ATF3-Cre-driven excision of floxed gene “X”) can be evaluated. The reviewer correctly alludes to the fact that one would not compare ATF3+/+:Xfl/fl mice with ATF3+/cre:Xfl/fl mice because the genetic background (i.e. the baseline) is different. The appropriate comparison is between ATF3+/cre:X+/+ and ATF3+/cre:Xfl/fl mice, as we have done substituting “PTEN” for “X”. Similarly one would not investigate the effect of any manipulation in C57Bl/6 mice with e.g. Swiss Webster mice as controls, since their genetic makeup differs.

In the revised Discussion: “ATF3 gene dosage effects (i.e. partial function in hemizygous mice) should not be an impediment to investigations of functions of other (floxed) genes or sequences provided the appropriate controls are used. There is simply a new baseline upon which other genetic manipulations (using ATF3-Cre-driven excision of floxed gene ”X”) can be evaluated. For this reason it is important that any investigation of the function of gene X compares ATF3+/cre:X+/+ with ATF3+/cre:Xfl/fl (or ATF3+/cre:X+/fl, for investigating possible haploinsufficiency of X), as we have done here substituting “X” with “PTEN”. Naturally, the effect of ATF3 haploinsufficiency on gene X expression and regulation would need to be determined beforehand. In this respect, the RNA sequencing data provided by Gey et al., 2016 on injured and uninjured facial motoneurons is a valuable starting point.“

The other part of this comment refers to ”gene-expression analyses“. We don't actually suggest gene expression analyses per se as a use for these mice anywhere in the MS (and so there is nothing to remove). That said, if the appropriate controls are used (as we lay out in the above paragraph and in our response to 1a), these mice would indeed be appropriate for such studies.

1d. The transgenic labeling procedure proposed here clearly has a pronounced effect on the regulation of the injury response in the labeled neurons, which would render their response abnormal as regards downstream genetic regulation.

Response: We apologize for the lack of clarity here. In none of our studies on anatomical or functional aspects of regeneration did we use reporter mouse crosses, so we can rule out reporter effects on the outcomes we report. We have clarified this in the methods section:

”To investigate the following anatomical and functional recovery outcomes we employed a transgenic line with only the Atf3 and Pten alleles altered (i.e. without reporter to avoid potential confounds associated with high tdtomato expression). Specifically, in the first set of experiments we compared ATF3+/+, ATF3+/cre and ATF3cre/cre mice in order to determine similarities to previous knockout models (e.g. Gey et al., 2016). In the second set we compared ATF3+/cre:PTEN+/+ mice with ATF3+/cre:PTENfl/fl in order to compare axotomy-induced PTEN deletion with virally-mediated PTEN deletion (Gallaher and Steward, 2018).”

1e. Thus, any experiments conducted to study genetic responses in these neurons would not be normal, and the responses to any modulatory interventions could not necessarily be viewed as normal. The authors do not mention any of this. Instead, the authors, make strong claims about the usefulness of the procedure in many contexts including to do gene expression analysis of injured neurons. This is not appropriate. The authors should remove the claims regarding gene expression analyses and functional relevance in the abstract and significance summaries. The technique is primarily useful for anatomical labelling, but far less so for genetic or functional studies. The authors should explicitly point this out so as not to mislead potential users of the technique. The authors should clearly address these points in the discussion and discuss the various ways in which AFT3 haploinsufficiency could alter data interpretation.

Response: The reviewer revisits the idea of “normal” or “baseline” and is correct in that comparisons between wild-type ATF3+/+ mice and ATF3+/cre:Xfl/fl mice would indeed be inappropriate because of the new baseline established by lacking one copy of the protein-encoding region of the ATF3 allele. What is considered “normal” is dictated by the background strain, which would be held constant in any experimental design. Again, what is normal for a C57Bl/6 mouse might not be normal for a B129 mouse, and so one would not investigate the role of a particular gene (by excising it) in Bl/6 mice while using B129 mice as (non-excised) controls.

2. Although the data presented in the figures are overall convincing, the authors should report statistical tests used and N in each figure legend.

Response: This has been done.

3. A major limitation of the study is the lack of axonal regeneration studies at later time points past day 2 post-injury. While the authors show reduction of functional recovery after ATF3 deletion, they do not study axonal regeneration past day 2, where they did not observe any difference. How can the authors explain this finding if they do not assess regeneration at later time points? Also, in the case of PTEN deletion where they find enhanced functional recovery, they do not study axonal regeneration past day 2. Additional time points measuring axonal regeneration are needed, or removal of claims regarding utility of this system for functional studies of nerve regeneration, as detailed in point 1 above.

Response: We have added a 3d timepoint in the ATF3 study, and find the expected differences between genotypes. Recall that the relationship between ATF3 (and PTEN) on axonal outgrowth is already established. We use our model to demonstrate its utility as new tool to excise sequences of interest upon axotomy. In this respect, it is important to show that the effects of ATF3 or PTEN deletion agree with previous studies, which we have done.

However, the reviewer's concern here can be addressed by us clarifying the utility of each type of outcome used in studies of peripheral nerve regeneration more generally: in our study it is assessed using three separate complementary techniques which ask slightly different questions. We have made major revisions to the MS and figures (according to the logic presented below) in order to make this more clear.

In behavioural studies we ask how differences in genotype affect the rate of functional recovery, which can only be attributable to axonal regeneration. In this arm of the experiment we do indeed assess recovery at multiple timepoints - every other day until one month post-injury. This is clarified in the methods section:

“Both tests [pinch and grasp] were carried out every other day starting on the third post-operative day until the experimental endpoint (day 28).”

While the behavioural data clearly show differences between the proportions of animals which respond to pinch or which are able to grasp at any particular timepoint, the binary nature (yes or no) of the data do not give an idea of “extent” of regeneration in individual animals.

Terminal EMG experiments provide insight in this respect. CMAP amplitudes indicate differences in the degree of muscle reinnervation, and latencies are a reflection of the maturity of regenerated axons.

From the Methods section: “We carried out terminal electromyographical experiments in order to understand the roles of ATF3 and PTEN in the extent of neuromuscular recovery following sciatic nerve injury. Compound muscle action potential (CMAP) amplitudes were used to indicate differences in the degree of muscle reinnervation, and thresholds and latencies were used to indicate the maturity of regenerated axons.”

Both of the above techniques unequivocally demonstrate deficiencies in functional regeneration in mice lacking one or both copies of ATF3, and enhanced functional recovery of motor function in mice lacking PTEN (on the ATF3 hemizygous background). In the former case we can rule out effects of compensation by uninjured axons since ATF3 is only upregulated in injured neurons; In the latter we can make the same conclusion since PTEN excision is restricted to injured neurons.

The anatomical studies ask a different question: How soon following injury can differences in axonal regeneration (already demonstrated functionally) be detected? In mice lacking PTEN the difference is already detectable at 2d post-injury, and so the question has been answered. In mice lacking ATF3 (one or both copies), the difference is not yet apparent at 2d post injury, but exists nonetheless, and we now show as much 3d post-lesion. We point out in the MS that this parallels findings on short-term in vitro experiments in which ATF3+/+ and ATF3-/- DRG neurons were treated with factors found in degenerating peripheral nerve (such as NGF): no differences in neurite outgrowth were observed (Gey et al., 2016). It is true that longer timepoints (in vitro or in vivo) might reveal at what point differences do become detectable. Such experiments, while providing additional information, have no bearing on the overall interpretation of our data - that regeneration is delayed in ATF3-deficient mice, and enhanced in PTEN-deficient mice.

By way of clarification, we have re-worded paragraphs in the results section:

“The above data unequivocally demonstrate delayed functional recovery (which can only be attributable to regeneration of injured axons given the absence of ATF3 expression in uninjured neurons) in ATF3-deficient mice. We then asked when differences in regeneration could be discerned between genotypes anatomically. To this end we assayed axonal regeneration along the sciatic nerve 2 and 3 days after crush by taking a single section (one that lacked sectioning artifacts like tears or folds) and imaged through its full 20um depth for analysis. Two days after sciatic nerve crush, cumulative axon density was not yet different between ATF+/+, ATFcre/+, and ATFcre/cre groups (Fig. 4A,B). Three days after sciatic nerve crush a significant difference was detected in cumulative axonal density at 2-4mm distal to the injury site between ATF+/+ and ATFcre/cre groups (p=0.0200), and the ATF+/cre group tested positive as a significant intermediary between groups (p=0.0108) (Fig. 4C,D). These anatomical results further support our findings that loss of one or both copies of the ATF3 allele diminishes regeneration after PNI.”

“Enhanced functional recovery in PTEN deficient mice implies more robust axonal regeneration. We were again curious as to when axonal regeneration following ATF3-driven injury induced Pten excision might be detected histologically following sciatic crush injury. Six animals were used in each group and a single section (lacking sectioning artifacts, and imaged through its full 20um depth) was taken for analysis. Two days after sciatic nerve crush, axon density plotted as a function of distance was obviously increased in ATF3+/cre PTENfl/fl over that in ATF3+/cre PTEN+/+ mice, and the cumulative density at 2 mm was statistically greater (p=0.0008) (Fig. 7F,G,H). This demonstrates that the ATF3+/cre line is able to edit the genes of injured neurons with enough efficacy to produce anatomical differences in regeneration.”

Nevertheless, we are including a 3 day timepoint for the anatomical ATF3 study. Going beyond 3 days pushes the limits of this type of analysis because axons regenerate at differing rates, and the increasing spread of axonal density along the nerve increases variability to the point of statistical intractability. By 3 days, the fastest-growing axons will have extended about 6 mm from the crush site (Shin et al., 2014), beyond which the anatomy of the sciatic nerve's branches renders this type of analysis impractical. Here, the functional assays of regeneration are of far greater value.

4. If the authors wish to argue that their ATF3-related expression patterns in regenerating/sprouting and non-regenerating cell bodies are linked to regenerative ability, they must show expression of a panel of regeneration associate genes in some of the neuronal populations investigated.

Response: It should be remembered that the purpose of our study is to present a method by which genomic editing can be effected by axotomy-induced ATF3 promoter activity, and is not meant to be an investigation of the role of ATF3 in regeneration. The experiments we include on ATF3 function are necessarily confirmatory, but are nevertheless informative in that they establish similarities between our knock-in and others' knockout models (e.g. Gey et al., 2016), and between axotomy-induced PTEN deletion and virally-mediated PTEN deletion (Gallaher and Steward, 2018). Such validation is required to establish the new tool, and is a major strength of our study.

From the introduction: “A loss of ATF3 function has been shown to reduce the regeneration observed after a peripheral nerve injury (PNI) (Gey et al 2016). Similarly, ATF3 overexpression enhances axonal outgrowth (Seijffers et al 2007).”

Also: “The loss of PTEN function via viral Cre transfection induces a regenerative response in corticospinal neurons after a spinal cord injury (Liu et al 2010), a finding that has been reported in other central nervous system (CNS) axonal injury models (Park et al 2008). In the PNS, in which regeneration is more robust but still suboptimal, PTEN deletion has been shown to have modest augmentative effects on axonal regeneration (Gallaher & Steward, 2018). This allows for the unique opportunity to compare a pathology-dependent Cre upregulation strategy with a more traditional viral Cre transfection model.”

The relationship between capacity for PNS vs CNS regeneration and ATF3 expression (again, not the main point of this study) is also already well-established: In the regeneration-incompetent CNS, ATF3 induction by axotomy is meagre and/or transient (reviewed in Hunt et al., 2012). We have used the Cre construct to show that despite this, several populations of CNS neurons can be genetically accessed by axotomy.

To clarify this point we have modified the appropriate section of the Discussion:

“ATF3 is not only upregulated after PNI but also after CNS trauma including spinal cord injury (Huang et al 2007), albeit usually meagerly and/or transiently, rendering expression patterns difficult to reveal (reviewed in Hunt et al., 2012). As such we wanted to determine if CNS neurons could be accessed genetically via ATF3-cre-mediated recombination after a lateral spinal cord hemisection. We observed consistent recombination in four deep brain nuclei (rubrospinal, reticulospinal, vestibulospinal, and hypothalamic) and their projecting axons and in at least two ascending tracts (putatively spinocerebellar and spinothalamic).”

## References

[B1] Agarwal N, Offermanns S, Kuner R (2004) Conditional gene deletion in primary nociceptive neurons of trigeminal ganglia and dorsal root ganglia. Genesis 38:122–129. 10.1002/gene.20010 15048809

[B2] Broude E, McAtee M, Kelley M, Bregman B (1997) c-Jun expression in adult rat dorsal root ganglion neurons: differential response after central or peripheral axotomy. Exp Neurol 148:367–377. 10.1006/exnr.1997.6665 9398479

[B3] Clausen BE, Burkhardt C, Reith W, Renkawitz R, Förster I (1999) Conditional gene targeting in macrophages and granulocytes using LysMcre mice. Transgenic Res 8:265–277. 10621974 10.1023/a:1008942828960

[B4] Clements MP, Byrne E, Guerrero LFC, Cattin A-L, Zakka L, Ashraf A, Burden JJ, Khadayate S, Lloyd AC, Marguerat S (2017) The wound microenvironment reprograms Schwann cells to invasive mesenchymal-like cells to drive peripheral nerve regeneration. Neuron 96:98–114. 10.1016/j.neuron.2017.09.008 28957681 PMC5626803

[B5] Darlot F, Vinit S, Matarazzo V, Kastner A (2017) Sustained cell body reactivity and loss of NeuN in a subset of axotomized bulbospinal neurons after a chronic high cervical spinal cord injury. Eur J Neurosci 46:2729–2745. 10.1111/ejn.13737 28977718

[B6] Denk F, Ramer LM, Erskine EL, Nassar MA, Bogdanov Y, Signore M, Wood JN, McMahon SB, Ramer MS (2015) Tamoxifen induces cellular stress in the nervous system by inhibiting cholesterol synthesis. Acta Neuropathol Commun 3:74. 10.1186/s40478-015-0255-6 26610346 PMC4660723

[B7] Fernandes KJ, Fan DP, Tsui BJ, Cassar SL, Tetzlaff W (1999) Influence of the axotomy to cell body distance in rat rubrospinal and spinal motoneurons: differential regulation of GAP-43, tubulins, and neurofilament-M. J Comp Neurol 414:495–510. 10531542 10.1002/(sici)1096-9861(19991129)414:4<495::aid-cne6>3.0.co;2-s

[B8] Förstner P, Rehman R, Anastasiadou S, Haffner-Luntzer M, Sinske D, Ignatius A, Roselli F, Knöll B (2018) Neuroinflammation after traumatic brain injury is enhanced in activating transcription factor 3 mutant mice. J Neurotrauma 35:2317–2329. 10.1089/neu.2017.5593 29463176

[B9] Gallaher ZR, Steward O (2018) Modest enhancement of sensory axon regeneration in the sciatic nerve with conditional co-deletion of PTEN and SOCS3 in the dorsal root ganglia of adult mice. Exp Neurol 303:120–133. 10.1016/j.expneurol.2018.02.012 29458059 PMC5864562

[B10] Gey M, Wanner R, Schilling C, Pedro MT, Sinske D, Knoll B (2016) Atf3 mutant mice show reduced axon regeneration and impaired regeneration-associated gene induction after peripheral nerve injury. Open Biol 6:160091. 10.1098/rsob.160091 27581653 PMC5008009

[B11] Gomez-Sanchez JA, Pilch KS, Van Der Lans M, Fazal SV, Benito C, Wagstaff LJ, Mirsky R, Jessen KR (2017) After nerve injury, lineage tracing shows that myelin and Remak Schwann cells elongate extensively and branch to form repair Schwann cells, which shorten radically on remyelination. J Neurosci 13 37:9086–9099. 10.1523/JNEUROSCI.1453-17.2017 PMC5597985 28904214

[B12] Guenthner CJ, Miyamichi K, Yang HH, Heller HC, Luo L (2013) Permanent genetic access to transiently active neurons via TRAP: targeted recombination in active populations. Neuron 78:773–784. 10.1016/j.neuron.2013.03.025 23764283 PMC3782391

[B13] Harty BL, Monk KR (2017) Unwrapping the unappreciated: recent progress in Remak Schwann cell biology. Curr Opin Neurobiol 47:131–137. 10.1016/j.conb.2017.10.003 29096241 PMC5963510

[B15] Hayashi S, McMahon AP (2002) Efficient recombination in diverse tissues by a tamoxifen-inducible form of Cre: a tool for temporally regulated gene activation/inactivation in the mouse. Dev Biol 244:305–318. 10.1006/dbio.2002.0597 11944939

[B14] Heumann R, Lindholm D, Bandtlow C, Meyer M, Radeke MJ, Misko TP, Shooter E, Thoenen H (1987) Differential regulation of mRNA encoding nerve growth factor and its receptor in rat sciatic nerve during development, degeneration, and regeneration: role of macrophages. Proc Natl Acad Sci U S A 84:8735–8739. 2825206 10.1073/pnas.84.23.8735 PMC299621

[B16] Huang WL, George KJ, Ibba V, Liu MC, Averill S, Quartu M, Hamlyn PJ, Priestley JV (2007) The characteristics of neuronal injury in a static compression model of spinal cord injury in adult rats. Eur J Neurosci 25:362–372. 10.1111/j.1460-9568.2006.05284.x 17284176

[B17] Hunt D, Hossain-Ibrahim K, Mason MR, Coffin RS, Lieberman AR, Winterbottom J, Anderson PN (2004) ATF3 upregulation in glia during Wallerian degeneration: differential expression in peripheral nerves and CNS white matter. BMC Neurosci 5:9. 10.1186/1471-2202-5-9 15113454 PMC400733

[B18] Hunt D, Raivich G, Anderson PN (2012) Activating transcription factor 3 and the nervous system. Front Mol Neurosci 5:7. 22347845 10.3389/fnmol.2012.00007 PMC3278981

[B19] Indra AK, Warot X, Brocard J, Bornert J-M, Xiao J-H, Chambon P, Metzger D (1999) Temporally-controlled site-specific mutagenesis in the basal layer of the epidermis: comparison of the recombinase activity of the tamoxifen-inducible Cre-ERT and Cre-ERT2 recombinases. Nucleic Acids Res 27:4324–4327. 10.1093/nar/27.22.4324 10536138 PMC148712

[B21] Kouyoumdjian JA (2006) Peripheral nerve injuries: a retrospective survey of 456 cases. Muscle Nerve 34:785–788. 10.1002/mus.20624 16881066

[B22] Leone DP, Genoud S, Atanasoski S, Grausenburger R, Berger P, Metzger D, Macklin WB, Chambon P, Suter U (2003) Tamoxifen-inducible glia-specific Cre mice for somatic mutagenesis in oligodendrocytes and Schwann cells. Mol Cell Neurosci 22:430–440. 12727441 10.1016/s1044-7431(03)00029-0

[B23] Lesche R, Groszer M, Gao J, Wang Y, Messing A, Sun H, Liu X, Wu H (2002) Cre/loxP-mediated inactivation of the murine Pten tumor suppressor gene. Genesis 32:148–149. 11857804 10.1002/gene.10036

[B24] Liang G, Wolfgang CD, Chen BP, Chen TH, Hai T (1996) ATF3 gene. Genomic organization, promoter, and regulation. J Biol Chem 271:1695–1701. 8576171 10.1074/jbc.271.3.1695

[B25] Liu K, Lu Y, Lee JK, Samara R, Willenberg R, Sears-Kraxberger I, Tedeschi A, Park KK, Jin D, Cai B, Xu B, Connolly L, Steward O, Zheng B, He Z (2010) PTEN deletion enhances the regenerative ability of adult corticospinal neurons. Nat Neurosci 13:1075–1081. 10.1038/nn.2603 20694004 PMC2928871

[B26] Liu Q, Trotter J, Zhang J, Peters MM, Cheng H, Bao J, Han X, Weeber EJ, Bu G (2010) Neuronal LRP1 knockout in adult mice leads to impaired brain lipid metabolism and progressive, age-dependent synapse loss and neurodegeneration. J Neurosci 30:17068–17078. 21159977 10.1523/JNEUROSCI.4067-10.2010 PMC3146802

[B27] Madisen L, Zwingman TA, Sunkin SM, Oh SW, Zariwala HA, Gu H, Ng LL, Palmiter RD, Hawrylycz MJ, Jones AR, Lein ES, Zeng H (2010) A robust and high-throughput Cre reporting and characterization system for the whole mouse brain. Nat Neurosci 13:133–140. 10.1038/nn.2467 20023653 PMC2840225

[B28] Mason MRJ, Lieberman AR, Anderson PN (2003) Corticospinal neurons up-regulate a range of growth-associated genes following intracortical, but not spinal, axotomy. Eur J Neurosci 18:789–802. 10.1046/j.1460-9568.2003.02809.x 12925005

[B30] Noble J, Munro CA, Prasad VS, Midha R (1998) Analysis of upper and lower extremity peripheral nerve injuries in a population of patients with multiple injuries. J Trauma 45:116–122. 10.1097/00005373-199807000-00025 9680023

[B31] Park KK, Liu K, Hu Y, Smith PD, Wang C, Cai B, Xu B, Connolly L, Kramvis I, Sahin M, He Z (2008) Promoting axon regeneration in the adult CNS by modulation of the PTEN/mTOR pathway. Science 322:963–966. 10.1126/science.1161566 18988856 PMC2652400

[B32] Rodríguez-Martínez JA, Reinke AW, Bhimsaria D, Keating AE, Ansari AZ (2017) Combinatorial bZIP dimers display complex DNA-binding specificity landscapes. Elife 6:e19272. 10.7554/eLife.19272 28186491 PMC5349851

[B33] Sainsbury A, Schwarzer C, Couzens M, Fetissov S, Furtinger S, Jenkins A, Cox HM, Sperk G, Hökfelt T, Herzog H (2002) Important role of hypothalamic Y2 receptors in body weight regulation revealed in conditional knockout mice. Proc Natl Acad Sci USA 99:8938–8943. 10.1073/pnas.132043299 12072562 PMC124402

[B34] Seijffers R, Mills CD, Woolf CJ (2007) ATF3 increases the intrinsic growth state of DRG neurons to enhance peripheral nerve regeneration. J Neurosci 27:7911–7920. 10.1523/JNEUROSCI.5313-06.2007 17652582 PMC6672733

[B35] Song DY, Oh KM, Yu HN, Park CR, Woo RS, Jung SS, Baik TK (2011) Role of activating transcription factor 3 in ischemic penumbra region following transient middle cerebral artery occlusion and reperfusion injury. Neurosci Res 70:428–434. 10.1016/j.neures.2011.05.002 21616101

[B36] Stambolic V, Suzuki A, de la Pompa JL, Brothers GM, Mirtsos C, Sasaki T, Ruland J, Penninger JM, Siderovski DP, Mak TW (1998) Negative regulation of PKB/Akt-dependent cell survival by the tumor suppressor PTEN. Cell 95:29–39. 9778245 10.1016/s0092-8674(00)81780-8

[B37] Sun F, Park KK, Belin S, Wang D, Lu T, Chen G, Zhang K, Yeung C, Feng G, Yankner BA, He Z (2011) Sustained axon regeneration induced by co-deletion of PTEN and SOCS3. Nature 480:372–375. 10.1038/nature10594 22056987 PMC3240702

[B38] Tetzlaff W, Alexander SW, Miller FD, Bisby MA (1991) Response of facial and rubrospinal neurons to axotomy: changes in mRNA expression for cytoskeletal proteins and GAP-43. J Neurosci 11:2528–2544. 1831228 10.1523/JNEUROSCI.11-08-02528.1991 PMC6575511

[B39] Tsujino H, Kondo E, Fukuoka T, Dai Y, Tokunaga A, Miki K, Yonenobu K, Ochi T, Noguchi K (2000) Activating transcription factor 3 (ATF3) induction by axotomy in sensory and motoneurons: a novel neuronal marker of nerve injury. Mol Cell Neurosci 15:170–182. 10.1006/mcne.1999.0814 10673325

[B40] van den Brink SC, Sage F, Vértesy A, Spanjaard B, Peterson-Maduro J, Baron CS, Robin C, Van Oudenaarden A (2017) Single-cell sequencing reveals dissociation-induced gene expression in tissue subpopulations. Nat Methods 14:935. 10.1038/nmeth.4437 28960196

[B41] Wagner KU, Wall RJ, St-Onge L, Gruss P, Wynshaw-Boris A, Garrett L, Li M, Furth PA, Hennighausen L (1997) Cre-mediated gene deletion in the mammary gland. Nucleic Acids Res 25:4323–4330. 9336464 10.1093/nar/25.21.4323 PMC147032

[B42] Wang W, Liu R, Xu Z, Niu X, Mao Z, Meng Q, Cao X (2015) Further insight into molecular mechanism underlying thoracic spinal cord injury using bioinformatics methods. Mol Med Rep 12:7851–7858. 10.3892/mmr.2015.4442 26497545 PMC4758289

